# Hypothalamic GHRH

**DOI:** 10.1007/s11154-025-09951-y

**Published:** 2025-02-06

**Authors:** Carlos Dieguez, Miguel López, Felipe Casanueva

**Affiliations:** 1 Department of Physiology, CiMUS, University of Santiago de Compostela-Instituto de Investigación Sanitaria de Santiago de Compostela, Santiago de Compostela, 15782,, Spain; 2 CIBER Fisiopatología de la Obesidad y Nutrición (CIBEROBN), Santiago de Compostela, 15706, Spain; 3 Department of Medicine, University of Santiago de Compostela-Instituto de Investigación Sanitaria de Santiago de Compostela, 15782, Santiago de Compotela, Spain; 4 Complejo Universitario de Santiago de Compostela, Santiago de Compostela, 15706, Spain

**Keywords:** Growth hormone releasing hormone, Somatostatin, Ghrelin, Growth hormone, Leptin, Orexin, Neuropeptide Y

## Abstract

Despite initial discovery in pancreatic tumors, GHRH is a 44-amino acid peptide primarily expressed in the hypothalamus. Recent RNA sequencing clarifies GHRH expression: predominantly hypothalamic in humans, with some basal ganglia presence, while extending to additional central nervous system (CNS) regions in other species. GHRH binds to its G-protein coupled receptor (GHRHR) in the arcuate (ARC), ventromedial (VMH), and periventricular (PeN) nuclei of the hypothalamus to exert its effects. Notably, the highest non-brain expression is found in somatotroph cells of the pituitary, directly targeting growth hormone (GH) production. GHRH is the primary regulator of pulsatile GH secretion, counteracted by somatostatin. While early models proposed alternating GHRH/somatostatin bursts, others implicate somatostatin as the primary regulator of GH pulse timing. These models fail to fully explain species and gender differences, particularly regarding nutritional status. The discovery of ghrelin, acting via GHS-R1a on GHRH neurons, significantly advanced understanding of GH regulation. Ghrelin interacts intricately with GHRH, modulating its expression and neuronal activity. Ghrelin also exerts GHRH-independent GH stimulation and synergizes with GHRH. The crucial role of GHRH in GH regulation is demonstrated by its key involvement in the action of other GH regulators, such as leptin, neuropeptide Y (NPY), and orexins. However, these interactions have also revealed that the physiological effects of GHRH extend far beyond its canonical role as a GH secretagogue. In this context, GHRH is thought to be a key regulator of the sleep-wake cycle and may be involved in whole-body energy homeostasis. The objective of this review is to summarize the current knowledge on GHRH and to discuss the potential pleiotropic effect of this hypothalamic neuropeptide, far beyond its classical action as regulator of the somatotroph axis.

## Introduction

Despite decades of intensive research, Growth Hormone-Releasing Hormone (GHRH) was initially identified in human pancreatic tumors rather than hypothalamic extracts. Subsequent studies confirmed its predominant expression in the hypothalamus under physiological conditions. GHRH belongs to the secretin family of evolutionarily related hormones, part of a larger superfamily including the glucagon family of peptides [1–2].

GHRH, synthesized from prepro-GHRH, is processed into a 44-amino acid peptide, with its first 29 amino acids recapitulating full biological activity. It regulates growth hormone (GH) synthesis and secretion via cyclic AMP– Protein Kinase A (cAMP-PKA) and mitogen-activated protein kinase (MAPK) signaling pathways, enhancing pituitary transcription factor 1 (Pit-1) levels and subsequently increasing GH gene expression [1–5].

Recent RNA sequencing studies have localized GHRH expression predominantly to in the human hypothalamus, with some expression in the basal ganglia. In mice, single-cell RNA sequencing revealed Ghrh + co-expression with Trh in a glutamatergic population (Glu3) and with Gal in a GABAergic (GABA11) neuronal population in the arcuate nucleus of the hypothalamus (ARC) [6–8].

The GHRH receptor (GHRHR), a class B G protein-coupled receptor (GPCR), mediates GHRH’s biological effects. GHRHR mRNA is highly expressed in the ARC, ventromedial (VMH), and periventricular (PeN) nuclei of the hypothalamus, with highest non-brain expression in anterior pituitary somatotrophs. GHRH and GHRHR expression have also been demonstrated in various peripheral tissues [1–2, 7].

GHRH is the primary physiological regulator of pulsatile pituitary GH secretion, as evidenced by suppression of GH secretion upon GHRH gene silencing or anti-GHRH antibody administration. Genetically modified animals with mutations affecting GHRH, GHRHR, or GH genes exhibit similar phenotypes, illustrating the relevance of all three components [9–12]. Human studies using GHRH-antagonist drugs further support GHRH’s role in GH secretion [13–15]. Spontaneous GH secretion and GH responses to pharmacologic stimuli of GH release such as arginine, L-dopa, insulin hypoglycemia, clonidine, or pyridostigmine were suppressed following administration of a specific GHRH-Antagonist [13–15]. While early rodent studies suggested alternating hypothalamic GHRH and somatostatin burst as primary drivers of GH pulsatility, other data implicated somatostatin as the key regulator of secretory timing and amplitude, with tonic GHRH secretion [16–19]. Species-specific differences, particularly regarding nutritional status impact, highlight the need for more adequate experimental models to address these complexities.

### Growth hormone short-loop feedback

It is well-established [20] that GH secretion is significantly influenced by a short-feedback loop mediated by both GH and insulin-like Growth factor 1 (IGF-1). Evidence from various experimental models suggests that while elevated GH levels decrease Ghrh mRNA levels in ARC neurons, the low GHR expression in these cells (< 10%) [21, 22] indicates an indirect effect mediated through other neuronal populations. Neuropeptide Y (NPY)-containing neurons within the ARC, many of which express Ghrh mRNA [21, 22], are potential candidates. Notably, decreased NPY mRNA levels in these neurons following hypophysectomy are reversed by GH administration. These findings suggest a crucial role for NPY neurons in mediating GH autofeedback [21, 22]. However, direct evidence to support this hypothesis remains elusive.

**Fig. 1:**
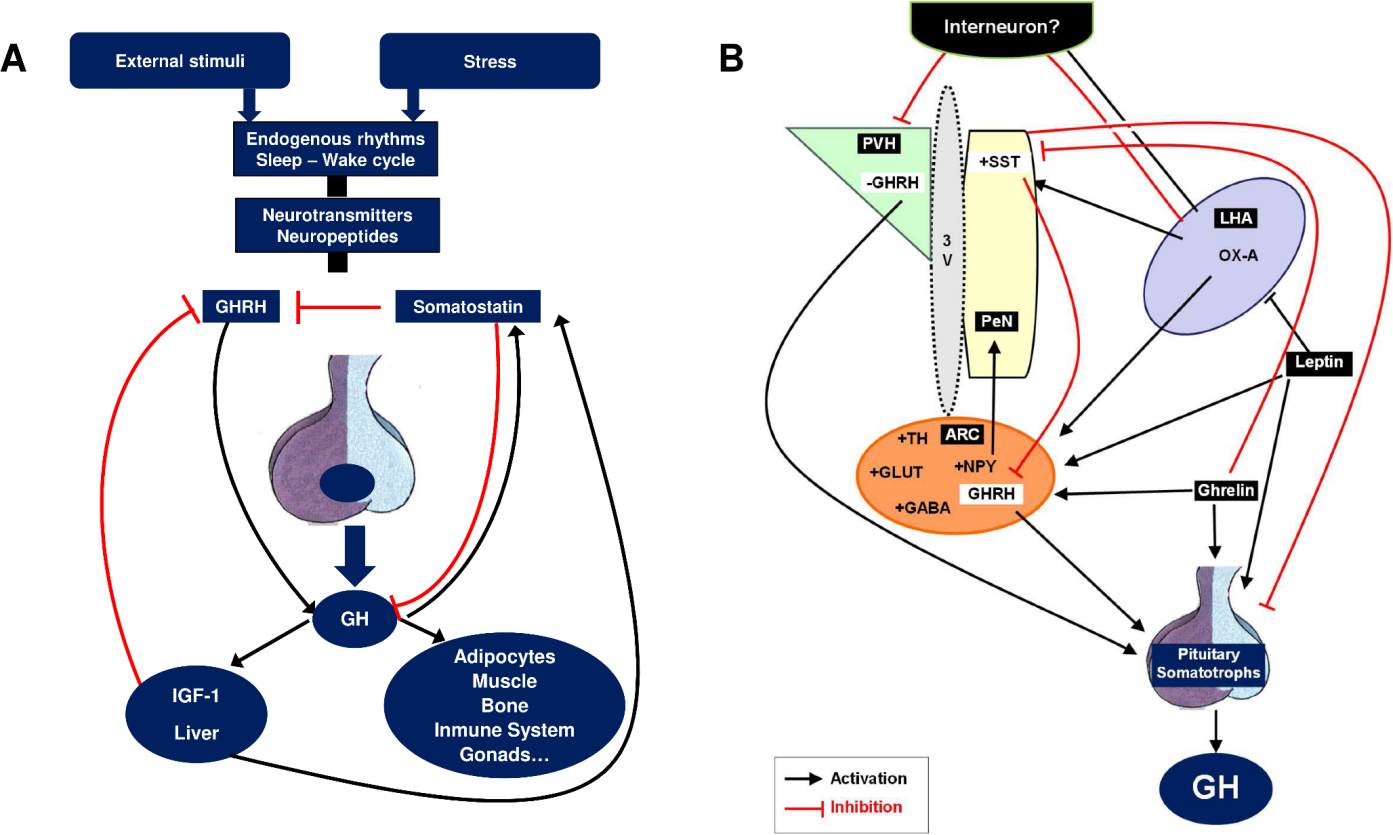
(A) Conceptual framework of the key hypothalamic pathways involved in the regulation of GH secretion, focusing on the short-loop feedback exerted by GH and the long loop feedback of IGF-1. (B) Mechanisms by which three key signals involved in energy homeostasis and sleep-wake cycle such as ghrelin, leptin and orexin A (OX-A) influence GH secretion secretion.

Conversely, the GHR is abundantly expressed in SST neurons of the PeN (> 70%). Furthermore, GH administration induces significant depolarization in many of these neurons. However, GHR ablation in these cells only elicits substantial changes in GH levels when performed concurrently with IGF-1 receptor ablation [23]. This suggests a shared mechanism through which both GH and IGF-1 act upstream to influence hypothalamic-pituitary-GH axis activity. Importantly, GHR ablation in tyrosine hydroxylase (TH)-expressing neurons leads to increased GH amplitude, IGF-1 levels, and animal growth [24]. This highlights the critical role of these neurons in the short-loop feedback mechanism (Fig. 1A).

### Ghrelin’s integration into GH regulation

The discovery of ghrelin as a potent GH secretagogue significantly advanced our understanding of GH regulation, previously viewed through the simplified GHRH-SST paradigm. Ghrelin interacts with GHRH in several ways: a) Ghrelin receptor 1a (GHS-R1a) is expressed in GHRH neurons of the ARC.b) GHS-R1a agonists increase c-Fos expression and neuronal firing in these neurons. c) Partial GHS-R1a silencing in these neurons reduces GHRH expression, while overexpression increases it. d) Reduced GHS-R1a expression is associated with decreased c-Fos and GHRH expression, and diminished GH responses to ghrelin. Notably, these changes correlate with decreased circulating GH and IGF-1 levels in female, but not male, rats. These findings underscore the intricate relationship between ghrelin and GHRH [25–31]. While ghrelin stimulates GH secretion independently of GHRH, acting synergistically with it in vivo [25–29], the precise mechanism remains unclear. Bowers *et al.* proposed a synergistic effect mediated by an unknown neuropeptide (U-factor) [32]. A more widely accepted mechanism involves ghrelin acting as a functional SST s antagonist in hypothalamic GH secretion regulation [29]. However, the exact mechanism remains elusive.

Ghrelin administration in humans increases both circulating GH and SST levels, suggesting that hypothalamic, but not peripheral, SST production is involved in GH secretion regulation [33]. Direct ghrelin action on hypothalamic somatostatin neurons appears unlikely, due to the lack of GHS-R1a expression in these cells and the absence of ghrelin-induced in vitro SST secretion. Assessment of SST levels in the hypophysial portal blood of conscious sheep following ghrelin agonist administration revealed increased GHRH pulse frequency without changes in somatostatin pulse frequency or amplitude [17]. Despite evidence suggesting ghrelin’s action as a functional SST antagonist, the underlying neuronal network remains unknown (Fig. 1B). Indirect evidence points towards an AMP-activated protein kinase (AMPK)-dependent mechanism mediating both the GH response to GHRH and ghrelin, resembling certain aspects of the ghrelin-orexigenic effect [34, 35].

### Leptin and GHRH

Leptin and GHRH play pivotal roles in the regulation of energy and metabolic homeostasis. As aforementioned, GHRH is predominantly expressed in the ARC, which is a region critical for maintaining energy balance. Given the significant impact of GH on glucose and lipid metabolism, it was hypothesized that mechanisms governing energy homeostasis might influence the hypothalamic-GH secretory axis, a concept further supported by the discovery of leptin. Experimental evidence has demonstrated leptin’s physiological influence on GH secretion. Intracerebroventricular (icv) administration of leptin antiserum resulted in reduced spontaneous GH secretion in normal rats [4]. Notably, while leptin did not alter GH secretion in normally fed rats, it reversed the inhibitory effects of fasting on GH secretion and enhanced responses to GHRH and GHRP-6 in fasted rats [36–38]. These findings underscore leptin’s role as a critical mediator in the regulation of GH secretion.

Further investigations into SST mRNA levels in PeN neurons of leptin-treated rats yielded unexpected results compared to in vitro studies. In vivo leptin administration increased SST mRNA levels; an effect mediated by elevated GH acting through GH receptors on these neurons. Furthermore, both leptin administration and passive immunization with SST antiserum restored GH secretion in food deprived rats [38]. The precise mechanism by which leptin affects GHRH and SST, whether directly or via NPY neurons, remains to be elucidated and warrants further investigation [39].

Beyond its hypothalamic actions, leptin receptors (LEPR) are expressed in somatotroph cells, where they exert a well-documented stimulatory effect [40, 41]. Notably, specific deletion of the LEPRb long-isoform signaling in somatotropes significantly reduces GH secretion, GH protein and mRNA levels, GHRH receptor binding, GHRH receptor mRNA levels, and leads to obesity [42]. However, the relevance of these findings to humans are unclear since the long-isoform receptor of leptin is present in fetal pituitaries and in pituitary tumors developed during postnatal life, but absent in pituitaries obtained from normal subjects postnatally [36].

Further human studies were undertaken to investigate the role for leptin in regulating GH secretion and its adaptation to changes in nutritional status, such as fasting. A primary characteristic of fasting is a decrease in leptin levels, signaling the hypothalamus regarding the availability of energy stores within adipocytes [36]. Consequently, spontaneous GH secretion is elevated in short-term fasted individuals. In contrast to findings in rodents, leptin administration fails to produce significant changes in spontaneous GH secretion in humans [43]. The reasons for this discrepancy remains unclear, but it is important to note that: a) Fasting effects differ between species: unlike humans, fasting suppresses GH secretion in rodents. b) Stress response varies: short-term fasting (e.g., 48 h) is considerably more stressful in mice than in humans, due to lower adipose tissue reserves [36]. Furthermore, children with leptin receptor mutations, but not those with leptin deficiency, exhibit early growth delay with subnormal concentrations of GH, IGF-I, and insulin-like growth factor binding protein 3 (IGFBP-3) [44]. This finding emphasizes the importance of leptin signaling in human GH regulation.

In summary, leptin plays a pivotal role in regulating the GH axis, (Fig. 1B), akin to its roles in other endocrine axes like those governing gonadal and thyroid functions, particularly evident in states of leptin deficiency, such as fasting. This mirrors its well-established impacts on body weight and energy homeostasis, which are starkly pronounced in leptin-deficient states, but less so in normal or obese individuals.

### Orexins and GHRH

In addition to regulating GH secretion and metabolic status, growth GHRH plays a role in promoting non-rapid eye movement (non-REM) sleep across various species through a mechanism independent of GH action [45]. Conversely, it also stimulates rapid eye movement (REM) sleep, but in this case, its effects are mediated by GH [45]. Given that sleep profoundly influences spontaneous GH secretion, considerable research attention has focused on signals such as orexins, involved in both GH regulation and sleep patterns [46, 47].

Over the past three decades, studies have highlighted the pivotal role of orexins in regulating the sleep-wake cycle [46, 47]. Given the link between ultradian GH secretion and the sleep-wake cycle, investigating the interplay among orexins, GHRH, and GH became a natural query. Orexin deficiency is notably associated with narcolepsy in most mammals, including humans, and these individuals exhibit an abnormal GH secretion profile characterized by elevated GH levels during daylight hours and reduced levels at night [48, 49]. However, their GH responses to GHRH remain normal. Additionally, these patients often present with an increased body mass index and other metabolic abnormalities [48, 49].

Further research into rodents has helped to uncover the underlying mechanisms. It is now established that exogenous administration of orexin-A (OX-A) inhibits spontaneous GH secretion in normal rats without altering GH responses to GHRH [50]. This effect does not correlate with changes in mRNA levels in GHRH neurons in the ARC. Instead, OX-A exhibits a clear stimulatory effect of NPY neurons of the ARC (which stimulates SST in the PeN, leading to GH inhibition) and a inhibitory effect on GHRH neurons located in the parvocellular region of the paraventricular nucleus of the hypothalamus (PVH), which project axons to fenestrated capillaries of portal vessels in the median eminence (ME), indicating a specific targeting of orexins on these neurons [51–53]. Considering that this latter neuronal population is involved in sleep-cycle regulation, it is tempting to speculate whether the inhibitory action of OX-A on REM sleep could be mediated, at least in part, by decreasing GHRH mRNA expression in the PVH. Future studies with selective suppression of these neurons are essential to elucidate whether these less-studied GHRH-expressing neurons are involved in sleep-associated GH surges.(Fig. 1B).

### Concluding remarks

In contrast to other hypothalamic hypophysiotropic hormones, GHRH expression is highly localized to specific neuronal populations in the human brain. The evolutionary basis for its more widespread expression in other species remains to be elucidated. Consistent with its restricted expression pattern, the distribution of the GHRH receptor is also more confined compared to other similar releasing hormones. Since its identification, accumulating evidence has consistently underscored the pivotal role of GHRH GH secretion across diverse animal species. Despite its profound effects on GH synthesis and secretion, GHRH has not been widely adopted as a standard therapeutic intervention for GH-deficient patients. Intriguingly, GHRH has also been implicated in the regulation of the sleep-wake cycle and metabolic homeostasis, through its interaction with other hypothalamic neuropeptide systems, such as orexins. It remains to be determined whether these diverse processes are regulated by the same GHRH-expressing neuronal population or by distinct subsets. Future investigations employing advanced techniques, such as single-cell and spatial transcriptomics, followed by comprehensive functional characterization, are eagerly anticipated. These studies will likely provide crucial insights into the molecular and cellular mechanisms underlying GHRH’s multifaceted roles in neuroendocrine regulation and potentially uncover novel therapeutic targets for disorders of GH secretion, sleep disturbances, and metabolic dysregulation.

## Data Availability

No datasets were generated or analysed during the current study.

## Abbreviations

AMPK: AMP-activated protein kinase
GHRH: Growth hormone releasing hormone
CNS: Central nervous system
GHRHR: GHRH-Receptor
GH: Growth hormone
GHR: Growth hormone receptor
GHS-R1a: GH-secretagogue receptor 1a
icv: Intracerebroventricular
IGF-1: Insulin-like growth factor 1
IGFBP-3: Insulin-like growth factor binding protein 3
LEPR: Leptin receptor
NPY: Neuropeptide Y
OX-A: orexin-A
cAMP-PKA: Cyclic AMP– Protein Kinase A
MAPK: Mitogen-activated protein kinase
GPCR: G protein-coupled receptor
SST: Somatostatin
ARC: Arcuate Nucleus of the hypothalamus
PeN: Periventricular Nucleus of the hypothalamus
Pit-1: Pituitary transcription factor 1
PVH: Paraventricular hypothalamus of the hypothalamus
ME: Median Eminence
VMH: Ventromedial nucleus of the hypothalamus
